# Fluid Intake of Pregnant and Breastfeeding Women in Indonesia: A Cross-Sectional Survey with a Seven-Day Fluid Specific Record

**DOI:** 10.3390/nu8110651

**Published:** 2016-11-22

**Authors:** Saptawati Bardosono, Damar Prasmusinto, Diah R. Hadiati, Bangun T. Purwaka, Clementine Morin, Rizki Pohan, Diana Sunardi, Dian N. Chandra, Isabelle Guelinckx

**Affiliations:** 1 Department of Nutrition, Faculty of Medicine, Universitas Indonesia, Jakarta 10430, Indonesia; diana_sunardi@yahoo.com (D.S.); diannovitach@yahoo.com (D.N.C); 2 Department of Obstetrics and Gynecology, Faculty of Medicine, University of Indonesia, Jakarta 10430, Indonesia; masdamar@yahoo.com; 3 Department of Obstetrics and Gynecology, Faculty of Medicine, Universitas Gadjah Mada, Yogyakarta 55281, Indonesia; rumekti@yahoo.com; 4 Department of Obstetrics and Gynecology, Faculty of Medicine, University of Airlangga, Surabaya 60112, Indonesia; b4ngun_tp@yahoo.com; 5 Hydration & Health Department, Danone Research, Palaiseau 91767, France; clementine.morin@danone.com (C.M.); isabelle.guelinckx@danone.com (I.G.); 6 R&D AQUA Group, Jakarta 12950, Indonesia; Rizki.Pohan@danone.com

**Keywords:** fluid intake, pregnant, breastfeeding, hydration, Indonesia, seven-day fluid record

## Abstract

During pregnancy and lactation, the adequate intake (AI) for total water intake is increased. This cross-sectional survey aimed to assess Total Fluid Intake (TFI; sum of drinking water and all other fluids) of 300 pregnant and 300 breastfeeding women in Indonesia. A seven-day fluid specific record was used to assess TFI. Mean TFI of pregnant and breastfeeding women were 2332 ± 746 mL/day and 2525 ± 843 mL/day, respectively. No significant difference in TFI between pregnancy trimesters was observed, while TFI of women breastfeeding for 12–24 months postpartum (2427 ± 955 mL/day) was lower than that of the two other groups (0–5 months: 2607 ± 754 mL/day; 6–11 months: 2538 ± 807 mL/day, respectively). Forty-two and 54% of the pregnant and breastfeeding subjects, respectively, did not reach the AI of water from fluids. These AI were actually known by only 14% and 23% of the pregnant and breastfeeding subjects. However, having the knowledge about the AI did not increase the odds of reaching the AI. Concluding that a high proportion of the pregnant and breastfeeding subjects did not reach the AI of water from fluid, it seems pertinent to further assess the fluid intake, as well as their hydration status, in other countries.

## 1. Introduction

During pregnancy, the water balance is altered due to an accretion in total body water. This total body water accretion measured by deuterium or antipyrine tracers is on average 7–8 L. For a gestational weight gain of about 12.5 kg, this total water gain is at term distributed in the fetus (2414 g), placenta (540 g), amniotic fluid (792 g), blood-free uterus (800 g), mammary gland (304 g), blood (1.267 g), and extracellular fluid (1.496 g) with no edema or leg edema [1]. Investigators found that the total body water accretion was positively correlated with birth weight [2,3] and the amount of amniotic fluid is a predictor of fetal well-being [4]. However, a direct association between total fluid intake, water intake or intake of any other fluid type and pregnancy outcome is rarely investigated. In an animal study vasopressin, a key player in water homeostasis was associated with a risk to develop preeclampsia; however, this remains a preliminary finding [5]. Pregnant women with bacteriuria have an increased susceptibility to pyelonephritis [6]. Since an increased water intake seems to prevent recurrent urinary tract infections in non-pregnant women [7], it seems relevant to evaluate this, in the future, in pregnant women. The same applies for constipation, which is frequent during pregnancy (>30%) because of hormones and dietary factors [8]. A diet rich in fiber and an increased fluid intake is recommended; however, this is rather a practice-based than evidence-based recommendation [8]. While the physiological changes in body water content during pregnancy are known, the scientific literature on the consequences of inadequate hydration and the benefits of increased water intake remains scarce and superficial.

During lactation, the water balance is also altered. Through breast milk, composed of 87% water, breastfeeding women have an additional water loss of on average 700 mL/day at eight weeks postpartum [9,10]. To ensure the accretion in total body water during pregnancy and the compensation of the additional water loss via breast milk during lactation, the requirements for total water intake (TWI, water originates from fluids and food moisture) are increased during pregnancy and lactation [11].

The reference values of TWI for children, adolescents and adults are adequate intakes (AI) based on observed intakes. However, to the best of our knowledge, only one survey that aimed to assess total fluid intake (sum of drinking water and all other beverages) of pregnant and breastfeeding women was published. This survey was performed in Mexico and showed that a sample of 153 pregnant and 155 breastfeeding women drank on average 2.62 L/day and 2.75 L/day, respectively [12]. The Institute of Medicine (IOM) based the reference values of TWI for pregnant and breastfeeding women on the median TWI observed in National Health And Nutrition Examination Survey III (NHANES III). The AI was set at 3.0 L/day for pregnant women and 3.8 L/day for breastfeeding women, which is an increase of 0.3 L/day and 1.1 L/day, respectively, compared to non-pregnant/non-breastfeeding women [13]. In 2010, the European Food Safety Authorities (EFSA) had no European data available on observed water intakes of pregnant women. Since energy intake during pregnancy increases by 300 kcal/day, they recommend pregnant women to increase TWI by 300 mL/day compared to non-pregnant women. This resulted in an AI of TWI established at 2.3 L/day. For breastfeeding women, the EFSA established the AI of TWI also based on theoretical reasoning: In order to compensate for the water loss through breast milk, water requirement needs to increase by 700 mL/day compared to non-breastfeeding women [14]. In Indonesia, observed intakes of water among pregnant and breastfeeding women were also lacking. Like the EFSA, the Indonesian Ministry of Health consequently built recommendations based on the theoretical relationship between water intake and energy intake, meaning that for each kcal of energy intake, 1 to 1.5 mL of water needs to be consumed [15]. Pregnant women are therefore advised to add 300 mL/day of water to the AI of 2.3 L/day recommended to non-pregnant women. Breastfeeding women are recommended to increase TWI by 800 mL/day during the first six months postpartum and by 650 mL/day after six months postpartum.

To address the lack of available intake data, the primary aim of this cross-sectional survey was to assess the intake of drinking water and all other beverages of a sample of pregnant and breastfeeding women representative of three large cities in Indonesia. The secondary aim was to assess the knowledge about the requirement of fluid intake during pregnancy and lactation.

## 2. Materials and Methods

### 2.1. Survey Sampling and Protocol

This cross-sectional survey was conducted in Jakarta, Yogyakarta and Surabaya, representing Java Island in Indonesia. Data collection was performed from January to April 2014. All households with pregnant and breastfeeding women under the selected maternity clinic in each of the study areas were eligible for recruitment. A stratified sampling technique was applied: pregnant women were stratified into three strata, i.e., first, second and third trimester; the breastfeeding women into three strata, i.e., 0–5, 6–11 and 12–24 months post-partum. The study aimed to recruit 600 subjects in total, with 200 subjects per study location (i.e., 100 pregnant and 100 breastfeeding women).

All eligible subjects were given oral and written information about the study objectives and protocol. If willing to participate, a written informed consent was obtained. The study was approved on 23 December 2013 by the Ethics Committee of the Faculty of Medicine Universitas Indonesia (number 783/H2.F1/ETIK/2012).

Thereafter, eligible women were screened for the inclusion and exclusion criteria. Study inclusion criteria were: having signed the informed consent, being pregnant or (exclusively and non-exclusively) breastfeeding, having an age above 18 years, living in the study area for at last one year, having a middle-level of socio-economic status (level B and C based on household expenditure according to AC Nielsen criteria). There were 3 additional inclusion criteria for pregnant women: meeting the stratification of the pregnancy trimester, having a singleton pregnancy and having no pregnancy complication such as hyperemesis gravidarum, hypertension or (gestational) diabetes based on interview and physical examination. Breastfeeding women also had to be apparently healthy, i.e., no acute or chronic diseases based on interview and physical examination. The exclusion criteria were being illiterate and having difficulty with oral communication.

This screening visit was performed by trained physicians and nutritionists in the selected maternity clinic. During the same visit, the seven-day fluid diary was delivered and explained to the subjects during a face-to-face interview. Each day, the same nutritionist visited the subject at home to collect the fluid record of the previous day and to provide a new record for the next day. During the first visit, pregnant women reported their pre-pregnancy weight and height, and lactating women their current weight and height. On day 8, the nutritionist completed the questionnaire on fluid intake knowledge with the subjects. The aim of these daily home visits was to maintain a high participation rate and to avoid subjects copying the previous day’s data into the next-day record. In total, 30 trained nutritionists were involved in the data collection. Each nutritionist was responsible for visiting a maximum of 10 subjects during the same period. This survey followed the same sampling method, protocol and fluid assessment as those taking part in the Liq.In^7^ surveys [16,17].

### 2.2. Assessment of Fluid Intake with a Seven-Day Fluid Specific Record

The fluid record was structured to collect the following detailed information on each drinking act in open spaces on the record: the hour of consumption, the type of fluid, the brand of fluid, the volume of the recipient from which the volume was consumed and the volume actually consumed. To assist the subjects in estimating the consumed volumes, the records were supported by a photographic booklet of standard containers of fluids.

All fluids recorded were classified accordingly: water (bottled water and tap water. The latter one is because of safety reasons boiled before consumption), hot beverages (coffee and tea), milk and derivatives, soft drinks (carbonated and non-carbonated sweetened drinks, carbonated and non-carbonated non-calorically sweetened drinks, ice-based. coconut-based, chocolate-based, and energy drinks), juices (fruit and vegetable-based drinks) and other beverages (traditional drinks, cereal drinks, herbal drink, soy bean milk, and others). Total fluid intake (TFI) was defined as the sum of volumes of all these categories. Any addition (e.g., sugar) to a fluid was not taken into account during the fluid classification. The water content of food was not assessed, and consequently not taken into account.

An adequate fluid intake was defined as a TFI above or equal to 80% of AI or TWI since 70% to 80% of TWI is assumed to come from fluids and 20%–30% from food moisture [14]. Consequently the cut-offs used in this analysis to identify an adequate intake were 2080 mL/day for pregnant women, 2480 mL/day for breastfeeding women 0–6 months postpartum and 2360 mL/day for women 7–24 months postpartum [15].

### 2.3. Knowlegde on Fluid Intake Recommendation

The knowledge of the participants about the AI was assessed with 2 multiple-choice questions. The two questions were “In your opinion, how much water should a pregnant woman drink to have an adequate fluid intake?” and “In your opinion, how much water should a breastfeeding woman drink to have an adequate fluid intake?” The possible answers to the first question were the following:

○ Minimally 600 cc less than recommended for non-pregnant woman
○ Minimally 300 cc less than recommended for non-pregnant woman
○ As recommended for non-pregnant woman
○ Minimally 300 cc more than the non-pregnant recommendation
○ Minimally 600 cc more than the non-pregnant recommendation
○ Others: _______________________________________________

The answers to the second question had a comparable format. Participants were requested to tick off only one answer.

### 2.4. Data Management and Analysis

Data were recorded daily using specific forms, and then checked, coded and entered into spread-sheets (SPSS version 21.0. SPSS Inc., Chicago, IL, USA). Subjects reporting a mean total daily fluid intake below 0.4 L/day or higher than 6 L/day, as well as subjects not completing all 7 days of the seven-day fluid record were excluded from the analysis. Data analysis was performed by using JMP (version 10, SAS Campus Drive, Cary, NC, USA). Continuous variables were presented as mean, standard deviation and percentiles and dichotomous variables as number and percentage. Statistical comparisons were performed by pregnancy trimesters, postpartum periods, and areas. The mean intakes are estimated values taking into account all consumers including non-consumers. A Wilcoxon paired test was used for multiple comparisons of continuous variable and chi-square for percentages. A *p*-value below 0.05 was considered significant.

## 3. Results

**Figure 1:**
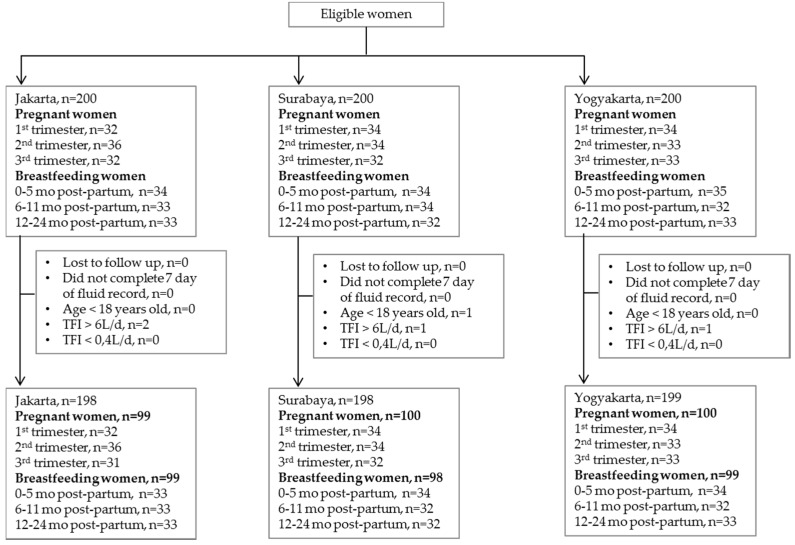
Flow chart of a cross-sectional survey recruiting pregnant and breastfeeding women in Indonesia.

The analysis was done on five hundred and ninety-five women of which 296 were breastfeeding and 299 were pregnant. A flow chart of the survey is presented in Figure 1 and the demographics of the subjects in Table 1. Of the subjects who completed the survey, most (56%) graduated from senior high school and 72% were housewives. In the pregnant sample, subjects were mainly overweight and obese (51%) with the highest proportion (60%) in Jakarta. Among the breastfeeding women, there was more normal weight than overweight or obese subjects (54% and 38%, respectively). Of the pregnant and breastfeeding women, 4% and 8%, respectively, were underweight.

### 3.1. Total Fluid Intake

Table 2 shows the mean and distribution in percentiles of TFI in both pregnant and breastfeeding women by study area, BMI classes, occupation and educational level. The mean TFI for pregnant and breastfeeding women was, respectively, 2332 ± 746 mL/day and 2525 ± 843 mL/day. In Jakarta, pregnant women had a higher TFI (2666 ± 681 mL/day) compared to those in Surabaya (2153 ± 732 mL/day; *p* < 0.0001) and Yogyakarta (2181 ± 717 mL/day; *p* < 0.0001). Among breastfeeding women, the same significant difference was observed between regions (Jakarta 2722 ± 897 mL/day; Surabaya 2 573 ± 899 mL/day; Yogyakarta 2280 ± 656 mL/day; *p* = 0.0006). No significant difference in TFI was found between pregnancy trimesters (*p* = 0.50), while the intake was different between the postpartum periods. Women breastfeeding for 12–24 months had a lower TFI (2427 ± 955 mL/day) than the two other groups of breastfeeding women (2607 ± 754 mL/day for 0–5 months postpartum; *p* = 0.005 and 2538 ± 807 mL/day for 6–11 months postpartum; *p* = 0.035). There was a significant difference in TFI between the BMI classess; however, this significant difference was only observed among pregnant women (*p* = 0.0047). There was no significant difference in TFI according to occupation or educational level among pregnant woman (*p* = 0.0938 and *p* = 0.4707, respectively). Similar observations were made among breasfeeding women (occupation *p* = 0.5887; educational level *p* = 0.8650).

### 3.2. Adherence to Indonesian Adequate Intake for Water from Fluids

Non-adherence to the AI was observed among 42% of the total sample of pregnant women. The lowest non-adherence to the AI was observed in Jakarta (18%) compared to 54% in Surabaya and 53% in Yogyakarta. No significant difference between stages of pregnancy was observed (1st trimester 46%; 2nd trimester 38%; 3rd trimester 42%; *p* = 0.50).

In the total sample of the breastfeeding women, 54% of women did not reach the AI for water from fluids. The highest non-adherence was observed in Yogyakarta (69%), then in Surabaya (56%) and the lowest proportion in Jakarta (37%). Non-adherence to the AI was the highest among women who were breastfeeding during 12–24 months (64%) compared to women breastfeeding during 0–5 months (50%) and those breastfeeding during 6–11 months (47%) (*p* = 0.035).

Table 3 shows the results of the assessment of the knowledge of the subjects on the AI of water from fluids. In the total sample, 14% and 23% of all subjects knew the AI for water from fluids of pregnant and beastfeeding women, respectively. Of the 47 pregnant women (16% of pregnant sample) who correctly identified the AI of water from fluids, 62% adhered to the AI and 38% did not. Of the 81 breastfeeding women (17% of breastfeeding sample) who knew the correct AI of water from fluids, 52% adhered to the AI of water from fluids for breastfeeding women and 48% did not. Having knowledge about the AI did not increase the odds of reaching the AI of water from fluids (pregnant women OR 1.18, 95% confidence interval 0.63–2.27; breastfeeding women OR 1.40, 95% confidence interval 0.84–2.34).

### 3.3. Consumption of Different Fluid Types

**Figure 2:**
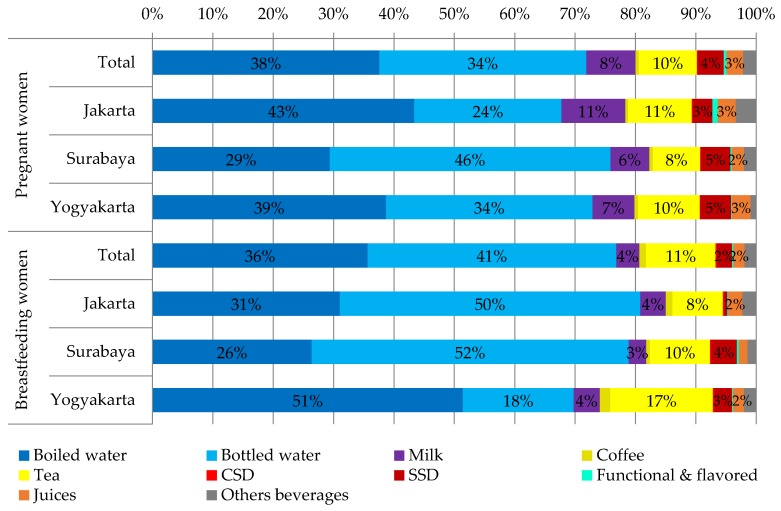
The contribution of different fluid types to total fluid intake of pregnant and breastfeeding women according to study area. Abbreviations: CSD: carbonated sweetened drinks; SSD: sugar sweetened drinks.

Table 4 shows the mean intake of the different fluid types by study area and Figure 2 the contribution of different fluid types to TFI. Among the different types of beverages, the highest intake in terms of volume and contribution to TFI was observed for water, especially boiled water for pregnant women in Jakarta and Yogyakarta (64% and 53% of drinking water, respectively) and bottled water for breastfeeding women in Jakarta and Surabaya (62% and 67% of drinking water, respectively). The second fluid type consumed the most were hot beverages with a daily intake ranging from 257 mL/day in Jakarta and 427 mL/day in Yogyakarta for breastfeeding women. For pregnant women, hot beverage intake was lower or similar to those of breastfeeding women, ranging from 181 mL/day in Surabaya and 293 mL/day in Jakarta. Soft drinks were the third most consumed beverages. Pregnant women had a significantly higher intake of soft drinks than brestfeeding women (115 ± 141 mL/day vs. 74 ± 116 mL/day, *p* < 0.0001). No significant differences was observed between study areas for pregnant women (*p* = 0.99), while for breastfeeding women, the intake of soft drinks ranged from 24 ± 61 mL/day in Jakarta to 123 ± 150 mL/day in Surabaya (*p* < 0.0001).

## 4. Discussion

To our knowledge, this is the first survey assessing the intake of water and all other beverages of a relatively large sample of Indonesian pregnant and breastfeeding women. The first finding of this survey was that the mean TFI of both the pregnant and breastfeeding women was 2.3 L/day and 2.5 L/day, respectively, which is different compared to published intake data among this target group. To the best of our knowledge, only two publications specifically focused on water or fluid intake among pregnant and breastfeeding women, and the mean TFI of a Mexican and Greek sample of pregnant woman was 2.6 L/day and 2.1 L/day, respectively [12,18]. The mean TFI of Mexican women breastfeeding during the first semester of lactation was 2.8 L/day [12]. Only hypotheses can be made to explain these intra-country differences. The methods used to assess the intake, climate and cultural factors could be possible explanations [19,20]. A second important observation is that compared to a Indonesian sample of non-pregnant and non-breastfeeding women previously investigated, the pregnant and breastfeeding women consumed on average only 0.26 L/day and 0.07 L/day more [16]. On one hand, this is similar to additional fluid intake (0.25 L/day) consumed by the pregnant Greek sample compared to the non-pregnant sample [18]. On the other hand, the additional TFI of the Mexican pregnant women (0.76 L/day) was much higher than that of non-pregnant Mexican women [12,16]. The Mexican women in the first semester of lactation increased their mean TFI with 0.96 L/day compared to the non-pregnant Mexican sample [12,16]. Even though the mean fluid increase of 0.26 L/day is relatively in line with the additional 300 mL/day of total water intake recommended by the Indonesian Ministry of Health, 42% of pregnant women in this sample still did not reach the AI of water from fluids. In the group of breastfeeding women, about half of the subjects (54%) did not adhere to the recommendation. Whether this increase was sufficient to cover the water requirements of the pregnant and breastfeeding women in this sample remains inconclusive due to lack of hydration biomarkers in the survey. A recent paper reported that urine color is also a valid marker of urine concentration among pregnancy and breastfeeding women, and consequently hydration status [21]. Using this hydration biomarker to have an indication of hydration status could be a suggestion for future large-scale surveys among this target.

The fluid type contributing the most to TFI among the pregnant and breastfeeding subjects was water (72%–77%). This was as expected since in a previous Indonesian adult sample drinking water contributed on average about 78% to TFI [17]. This is, however, an intake pattern specific to Indonesia. About 50% (1.44 L/day) of the water intake from fluids of the Greece pregnant women was drinking water and 33% (0.9 L/day) of the Mexican sample of pregnant women [12,18]. Among the Mexican sample of breastfeeding women, drinking water also contributed about 33% to TFI [12].

The observed difference in TFI between regions deserves a discussion. The pregnant and breastfeeding participants in Jakarta had a higher mean TFI and were consequently more likely to adhere to the AI than the participants in Surabaya and Yogyakarta. Since the socio-demographic characteristics of the participants were comparable at baseline, and since the climate, the method to assess fluid intake and period of data collection were comparable between regions, these factors could not explain the observed differences. In a national Total Diet Study, differences in nutrients and food intake were also observed, and differences in culture or habits was one of the suggested explanations [22]. The use of spices is, for example, different. Since capsaicin, a component of spicy food, can induce, via TRPV1, receptor thirst [23], this could be a potential explanation. That the regional differences were mainly related to a difference in water intake, and more specifically bottled or boiled water, could also suggest that water availability could be a factor influencing the intake of the participants. Intervention trials indeed showed that having water available stimulates consumption [24,25,26]. Besides water availability, education and having knowledge about the importance of water consumption was also identified as a key factor to effectively increase water intake [24,25,26]. In this survey, having the knowledge of the AI of water from fluids, however, did not increase the odds of adhering to the recommendations. This seems to be in line with previous evidence which indicated that one factor by itself (e.g., having the knowledge on AI) is not sufficient to induce a behavior, but a combination of multiple factors addressing the individual and their environment is required to maximize the chances for a behavior change [27]. A combination of factors could be providing nutritional education, ensuring access to water and providing them tools such as the urine color chart [21] to assess hydration status and/or measure fluid intake. Because many women are concerned about the health of their babies during pregnancy and are in frequent contact with their healthcare providers, pregnancy may be an especially powerful "teachable moment" for the promotion of healthy behaviors among women [28].

This survey does have limitations that we acknowledge. Data collection was performed in three cities, Jakarta. Surabaya and Yogyakarta, all three located on the Java Island. Since other nutritional surveys showed differences in nutritional intake between Indonesian provinces [22] and since ambient temperature and climate can be difference between the provinces, the findings of this study sample can therefore only be extrapolated to the pregnant and breastfeeding population of Java Island. Furthermore, this was a cross-sectional, and not a longitudinal survey in which subjects were followed from pre-pregnant status throughout pregnancy into the postpartum period. The comparison between pregnancy trimesters and between pregnant and breastfeeding should therefore be done with caution. The data collection was performed during one period of the year meaning that seasonality was not taken into account. However, since Java Island is situated around the equatorial line and the dry and rainy season do not differ in temperature, changes in fluid intake due to seasonality are expected to be limited. As mentioned previously, in the absence of hydration biomarkers, no conclusions can be drawn on the hydration status of the study sample.

Despite the limitations discussed above, this survey has several strengths and therefore contributed valuable information to the study of fluid consumption among specific target populations. Firstly, a large sample was recruited, with an equal distribution over pregnancy trimester and postpartum period. Moreover, the study was completed with a high compliance rate (i.e., >95%). Secondly, fluid intake was assessed used a seven-day fluid specific record, which is considered the reference method in nutritional assessment [29]. Moreover, this record was supported by a photographic booklet of standard containers, increasing the likelihood of having an accurate estimation of the consumption of the subjects. A potential risk of a dietary record is that the subject completes the record only the day after or even later, or that a subject copies the info recorded from one day to another day. Since the investigators visited the subject each day to check the completion, and recuperated the page of the fluid record, this risk was eliminated. In national surveys, a food frequency questionnaire, a 24 h dietary recall, or a mix of methods is more frequently used than a seven-day dietary record [30]. Since a 24 h recall tends to underestimate TFI [31], our results should be compared to other food surveys with caution.

## 5. Conclusions

To conclude, this survey indicated that a large proportion of this Indonesian pregnant and breastfeeding sample had an inadequate intake of water from fluids. Even though the evidence of potential positive health benefits of an increased water intake during pregnancy and lactation is currently limited, this finding suggests that the actions of midwives, general practitioners and other doctors to promote an increased water intake as part of a healthy lifestyle are pertinent.

## Figures and Tables

**Table 1:** General characteristics of the pregnant and breastfeeding women categorized by study area.

Variables	Total	Jakarta	Surabaya	Yogyakarta
**Age (years)**				
Pregnant women	28.5 (4.3)	29.2 (4.4)	28.5 (4.1)	27.8 (4.3)
Breastfeeding women	28.6 (4.0)	28.9 (4.2)	28.5 (3.9)	28.5 (3.8)
**Education Level**				
None	4 (1)	0 (0)	1 (1)	3 (2)
Elementary school	41 (7)	20 (10)	14 (7)	7 (4)
Junior high school	109 (18)	38 (19)	33 (17)	38 (19)
Senior high school	335 (56)	117 (59)	103 (52)	115 (58)
Higher education	106 (18)	23 (12)	47 (24)	36 (18)
**Working Status**				
Any job	3 (1)	1 (1)	2 (1)	0 (0)
Housewife	428 (72)	159 (80)	133 (67)	136 (68)
College student	2 (0)	0 (0)	0 (0)	2 (1)
Labor	4 (1)	2 (1)	1 (1)	1 (1)
Service	5 (1)	2 (1)	1 (1)	2 (1)
Para/medical profession	20 (2)	0 (0)	10 (5)	0 (0)
Education	5 (1)	1 (1)	2 (1)	2 (1)
Finance/Business	29 (5)	6 (3)	7 (4)	16 (8)
Government employment	10 (2)	3 (2)	3 (2)	4 (2)
Private sector employment	98 (16)	24 (12)	39 (20)	35 (18)
Other	1 (0)	0 (0)	0 (0)	1 (1)
**BMI Categories**				
Pregnant women ^1^				
Underweight	13 (4)	2 (2)	2 (2)	9 (9)
Normal weight	134 (45)	37 (37)	47 (47)	50 (50)
Overweight	110 (37)	37 (37)	41 (41)	32 (32)
Obese	42 (14)	23 (23)	10 (10)	9 (9)
Breastfeeding women				
Underweight	25 (8)	8 (8)	6 (6)	11 (11)
Normal weight	160 (54)	53 (54)	53 (54)	54 (55)
Overweight	76 (26)	29 (29)	25 (26)	22 (22)
Obese	35 (12)	9 (9)	14 (14)	12 (12)

Continuous data are presented as mean (SD) and dichotomous as *n* (%). ^1^ Body Mass Index (BMI) of pregnant women was calculated with pre-pregnancy weight.

**Table 2:** Total Fluid Intake (mL/day) of the pregnant and breastfeeding women categorized by study area, classes of body mass index, Occupation and Educational level.

Total Fluid Intake	*n* (%)	Mean (SD)	Percentiles						
			5	10	25	50	75	90	95
**Pregnant Women**									
Total	299 (100)	2332 (746)	1243	1436	1784	2229	2800	3307	3679
Jakarta	99 (33)	2666 (681)	1529	1784	2191	2721	3045	3643	3871
Surabaya	100 (33)	2153 (732)	1179	1343	1666	2000	2609	3298	3444
Yogyakarta	100 (33)	2181 (717)	1133	1441	1645	2039	2635	3129	3615
Underweight	13 (4)	1925 (535)	1129	1174	1607	1894	2195	2920	3119
Normal Weight	134 (45)	2210 (706)	1167	1416	1691	2129	2726	3142	3413
Overweight	110 (37)	2477 (771)	1315	1441	1876	2476	3053	3493	3857
Obesity	42 (14)	2469 (763)	1350	1606	1940	2356	2853	3567	4176
Housewife	186 (62)	2397 (779)	1183	1471	1795	2330	2900	3363	3861
Other occupation	113 (38)	2226 (678)	1272	1407	1726	2163	2693	3216	3473
Senior high school	163 (55)	2305 (751)	1226	1401	1750	2161	2833	3326	3580
Other educational level	136 (45)	2365 (740)	1313	1474	1837	2279	2791	3313	3850
**Breastfeeding Women**									
Total	296 (100)	2525 (843)	1491	1654	1971	2306	2901	3697	4357
Jakarta	99 (33)	2722 (897)	1463	1670	2024	2507	3240	3969	4581
Surabaya	100 (33)	2573 (899)	1599	1675	1952	2276	2979	3800	4404
Yogyakarta	99 (33)	2280 (656)	1476	1597	1907	2151	2476	3219	3519
Underweight	25 (8)	2430 (927)	1368	1460	1873	2266	2691	3559	5194
Normal Weight	160 (54)	2450 (778)	1582	1638	1910	2281	2777	3519	3942
Overweight	76 (26)	2655 (922)	1395	1647	2046	2438	3128	3840	4687
Obesity	35 (12)	2653 (871)	1718	1866	2000	2318	2957	4210	4643
Housewife	242 (82)	2530 (873)	1481	1638	1949	2285	2975	3764	4377
Other occupation	54 (18)	2500 (697)	1619	1724	2094	2410	2676	3324	3879
Senior high school	172 (58)	2522 (804)	1550	1661	1963	2306	2956	3709	4257
Other educational Level	124 (42)	2529 (897)	1433	1624	1979	2319	2825	3725	4396

**Table 3:** Assessment of the knowledge of pregnant and breastfeeding women on the Indonesian Adequate intake for water from fluids.

		How Much Water Should a Pregnant Woman Drink Daily?	How Much Water Should a Breastfeeding Woman Drink Daily?
Total women	Incorrect answer	514 (86)	455 (77)
	Correct answer	81 (14)	140 (23)
Pregnant women	Incorrect answer	252 (84)	240 (80)
	Correct answer	47 (16)	59 (20)
Breastfeeding women	Incorrect answer	262 (88)	215 (73)
	Correct answer	34 (12)	81 (17)

Data expressed as *n* (%).

**Table 4:** Total daily consumption of different fluid types (mL/day) in the pregnant and breastfeeding women by study area.

	Pregnant Women (*n* = 299)				Breastfeeding Women (*n* = 296)			
	Total	Jakarta	Surabaya	Yogyakarta	Total	Jakarta	Surabaya	Yogyakarta
**Water**	1676 (737)	1806 (681)	1633 (755)	1590 (761)	1939 (885)	2199 (922)	2029 (904)	1591 (707)
Boiled water	877 (880)	1156 (912)	633 (780)	844 (872)	900 (1028)	845 (1173)	679 (961)	1173 (875)
Bottled water	799 (895)	650 (808)	1001 (1000)	746 (837)	1040 (1109)	1354 (1130)	1351 (1174)	418 (686)
**Milk**	191 (181)	282 (213)	139 (142)	152 (143)	97 (142)	116 (146)	75 (124)	99 (151)
**Hot bev.**	237 (199)	293 (195)	181 (186)	236 (202)	319 (275)	257 (204)	271 (243)	427 (329)
Coffee	12 (46)	12 (44)	12 (47)	13 (47)	28 (69)	30 (68)	16 (52)	39 (83)
Tea	224 (188)	281 (186)	169 (171)	223 (191)	290 (256)	227 (190)	256 (237)	388 (302)
**Soft drinks**	115 (141)	115 (139)	113 (147)	116 (138)	74 (116)	24 (61)	123 (150)	76 (96)
CSD	3 (13)	6 (19)	2 (9)	2 (9)	6 (22)	7 (29)	6 (18)	5 (17)
SSD	101 (126)	86 (96)	105 (143)	112 (133)	62 (104)	13 (38)	108 (135)	66 (94)
Functional & flavored drinks	11 (38)	23 (60)	6 (19)	2 (15)	6 (24)	4 (21)	9 (32)	5 (17)
**Juices**	63 (85)	80 (94)	43 (74)	68 (82)	47 (93)	64 (107)	37 (83)	41 (87)
**Other bev.**	51 (90)	90 (123)	43 (74)	20 (38)	48 (83)	62 (90)	37 (78)	46 (78)

Data expressed as mean (SD); Abbreviations: bev.: beverages; CSD: carbonated sweetened drinks; SSD: sugar sweetened drinks.
